# Decrease in Secondhand Smoke Exposure in Work and Public Places among Adults in the Philippines: An Analysis of the Global Adult Tobacco Survey, 2009 and 2015

**DOI:** 10.3390/ijerph20021077

**Published:** 2023-01-07

**Authors:** Alissa C. Kress, Lazarous Mbulo, Carlen Stadnik, Rizalina Hemedez-Gonzalez, Evelyn Twentyman, E. Ulysses Dorotheo, Liping Pan

**Affiliations:** 1 Office on Smoking and Health, National Center for Chronic Disease Prevention and Health Promotion, Centers for Disease Control and Prevention, Atlanta, GA 30341, USA; 2 Oak Ridge Institute for Science and Education, Oak Ridge, TN 37830, USA; 3 Tobacco Advocacy Group, Philippine Pediatric Society, Quezon City 1101, Philippines; 4 Southeast Asia Tobacco Control Alliance, Bangkok 10300, Thailand

**Keywords:** secondhand smoke, tobacco smoke pollution, smoking, tobacco, Philippines

## Abstract

The implementation of several tobacco control policies in the Philippines may have contributed to a decrease in secondhand smoke (SHS) exposure. We examined changes in SHS exposure at work and in public places between 2009 and 2015 among adults aged ≥15 years and interpreted these results within the tobacco policy landscape in the Philippines. We analyzed the Philippines Global Adult Tobacco Survey 2009 and 2015 data. We examined marginal effects in logistic regression to get the adjusted prevalence of SHS exposure at five work and public places, controlling for selected characteristics. We calculated adjusted prevalence ratios and adjusted prevalence differences between 2009 and 2015. Adjusted prevalence of SHS exposure decreased from 2009 to 2015 by 19% (5.7 percentage points) at work, 45% (11.2 percentage points) in government buildings, 48% (3.2 percentage points) in healthcare facilities, 29% (8.2 percentage points) in restaurants, and 33% (19.9 percentage points) on public transportation. Although the prevalence of SHS exposure at work and in public places decreased significantly between 2009 and 2015, a substantial proportion of adults remain exposed to SHS. This study highlights the importance of continued implementation, enforcement, monitoring, and evaluation of tobacco control and prevention measures in the Philippines.

## 1. Introduction

Secondhand smoke (SHS) exposure, or the involuntary inhalation of tobacco smoke while not actively smoking, has been linked to lung cancer, heart disease, and stroke, along with various ill health effects among infants and children [1,2,3]. There is no known safe level of exposure to SHS [3]. Globally, more than one-third of adults are regularly exposed to SHS, and nearly 4 in 10 children have at least one smoking parent [4]. An estimated 1.2 million non-smokers die globally as a result of being exposed to SHS [5]. The prevalence of secondhand smoke exposure is particularly high in low- and middle-income countries (LMICs) [1].

In the Philippines, an LMIC in Southeast Asia, SHS exposure differs by setting. In 2009, over half of adults and approximately 19 million children were exposed to SHS in the home [6,7]; over 30% of adults who worked outside the home were exposed to SHS [6,8,9]. In public places, over half of adults were exposed to SHS on public transportation, over 30% in restaurants, approximately 25% in government buildings, and less than 10% in healthcare facilities [6,8]. By 2015, the prevalence of exposure to SHS among adults in any of those four public places had decreased by 17.2 percentage points [10].

**Figure 1:**
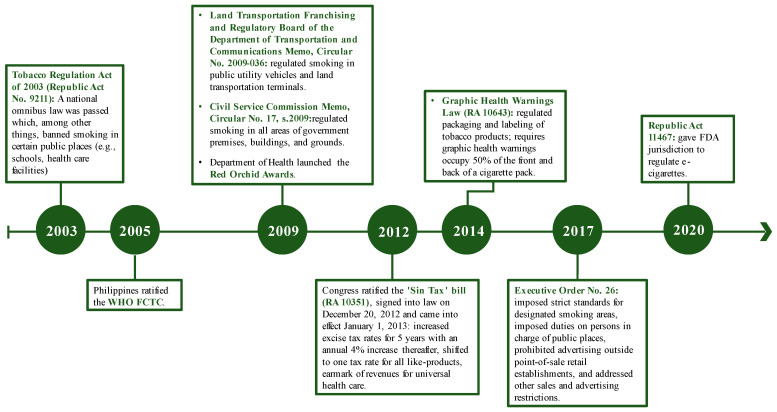
Timeline of selected tobacco control policies in the Philippines, 2003–2020 [11,12,15,16].

The Philippines has taken significant steps to address SHS exposure in the country, including becoming a party to the World Health Organization (WHO) Framework Convention on Tobacco Control (FCTC) in 2005 [11,12]. In support of Article 8 of the FCTC, which focuses on protection from exposure to tobacco smoke, WHO provides four policy recommendations: (1) implement 100% smoke-free environments, (2) enact legislation requiring all indoor workplaces and public places to be 100% smoke-free, (3) implement and enforce smoke-free legislation, and (4) implement educational strategies to reduce SHS exposure in homes [13]. Over the years, several notable tobacco control policies have been implemented in the Philippines, including laws banning smoking in certain public places [11,12,14]; increasing excise taxes on tobacco products and shifting to a uniform tax rate for cigarettes [11,15]; regulating packaging and labeling of tobacco products, including graphic health warning requirements [12,15]; and imposing standards for designated smoking areas and duties on people in charge of public places, as well as addressing other sales and advertising restrictions (Figure 1) [12]. As of December 2021, smoking was prohibited in some indoor public places and workplaces (e.g., government facilities, healthcare facilities, educational institutions), and most public transportation; however, designated smoking areas were still allowed in others (e.g., bars and nightclubs) [12].

Few published studies have assessed SHS exposure in the Philippines in the context of smoke-free policies. One study, published in 2013, used Philippines Global Adult Tobacco Survey (GATS) data collected four months after smoke-free regulations were expanded in 2009; it may have been too early to see the full effect [8]. Legislation introduced in subsequent years may also have affected SHS exposure since then. Our objective was to examine changes in SHS exposure at work and in public places between 2009 and 2015 among adults aged ≥15 years and discuss these changes within the broader tobacco policy landscape in the Philippines.

## 2. Materials and Methods

### 2.1. Data Source

We used data from two waves of GATS Philippines conducted in 2009 and 2015. Both surveys were implemented as nationally representative cross-sectional household surveys of adults aged 15 years or older with a total of 9701 respondents in 2009 (94.8% response rate) and 11,644 respondents in 2015 (92.1% response rate). Both survey waves used a global standardized methodology to collect data for systematic monitoring and tracking of tobacco use and other key tobacco control indicators in the country. The surveys collected information on demographic characteristics, tobacco use, cessation, SHS exposure, economics, and media, as well as knowledge, attitudes, and perceptions about tobacco use. A geographically clustered, multistage sampling methodology was used to divide the Philippines into Primary Sampling Units (PSUs) of randomly allocated barangays (a barangay is the smallest government administrative unit and loosely corresponds to a village or community, although some barangays are large and encompass several villages). PSUs were divided into segments, which were further divided into households. A random sample of households was selected for screening. Individual interviews were conducted among those aged 15 years or older, who were sampled from their household of residence [17]. Detailed information on GATS methodology is available elsewhere [18,19,20].

### 2.2. Measures

Smoke-free policies at work were assessed among respondents who reported working outside the home, either indoors or outdoors, using the question “Which of the following best describes the indoor smoking policy where you work: smoking is allowed anywhere, smoking is allowed only in some indoor areas, smoking is not allowed in any indoor areas, or there is no policy?” Respondents who indicated smoking is not allowed in any indoor areas were categorized as working at a workplace with a smoke-free policy.

The belief that SHS exposure causes serious illness in non-smokers was defined using the question “Based on what you know or believe, does breathing other people’s smoke cause serious illness in non-smokers?” with responses of “yes” and “no”.

SHS exposure at work was assessed among respondents who reported working outside the home, either indoors or outdoors. Those who reported seeing anyone smoke in indoor areas where they work in the past 30 days were categorized as having been exposed to SHS at work.

SHS exposure in various public places was defined using a series of questions. First, respondents were asked if they had visited government buildings, healthcare facilities, restaurants, or public transportation in the past 30 days. Those who indicated visiting a specific public place were asked if anyone smoked inside that public place in the past 30 days. Respondents who reported someone smoking inside the public place(s) they visited in the past 30 days were categorized as having been exposed to SHS at the place(s). Respondents who reported they “don’t know” if someone smoked inside the public place(s) were assumed to have not visited all parts and therefore, were not exposed to SHS [21]. In 2015 only, respondents were also asked about bars or nightclubs, universities, and schools or educational facilities.

Current tobacco smoking was defined using the question “Do you currently smoke tobacco?” Responses included daily, less than daily, not at all, don’t know, and refused. Respondents who answered “daily” or “less than daily” were categorized as currently smoking tobacco, while those who answered “not at all” were categorized as not currently smoking tobacco.

Sociodemographic characteristics included age group (15–24, 25–44, 45–64, and 65 years or older), sex (male, female), residence (urban, rural), wealth index (lowest, low, middle, high, highest quintile), education (no formal education, primary, secondary, higher than secondary), and occupation (employed, self-employed, student, homemaker, unemployed/retired). The wealth index is computed based on household ownership of assets such as electricity, flush toilet, fixed telephone, cell phone, television, and others. Based on the response to ownership of household assets, respondents were categorized into wealth quintiles ranging from lowest to highest [22].

### 2.3. Statistical Analyses

We used descriptive statistics to calculate the prevalence and 95% confidence intervals (CIs) of selected sociodemographic characteristics, tobacco use, smoke-free policies at work, and beliefs that SHS exposure causes serious illness in non-smokers among adults aged 15 years or older in the Philippines for 2009 and 2015. We then calculated prevalence and 95% CIs of SHS exposure at work and in various public places (government buildings, healthcare facilities, restaurants, and public transportation for both 2009 and 2015; bars or night clubs, universities, and schools or educational facilities for 2015 only) by sociodemographic characteristics, tobacco use, smoke-free policies at work, and believing that SHS exposure causes serious illness in non-smokers. Prevalence estimates were suppressed if the unweighted denominator was less than 25. We used *t*-tests to compare the difference in the unadjusted prevalence of SHS exposure between years. Finally, we examined the marginal effects in logistic regression to get the adjusted prevalence of SHS exposure at work, in government buildings, healthcare facilities, restaurants, and public transportation (for both 2009 and 2015), and adjusted prevalence ratios and adjusted prevalence differences between 2015 and 2009, controlling for selected sociodemographic characteristics, tobacco use, smoke-free policies at work, and beliefs that SHS exposure causes serious illness in non-smokers. The adjusted prevalence difference between years was considered statistically significant if the *p*-value was <0.05. We conducted analyses using SAS-callable SUDAAN v11.0.1 (RTI International, Research Triangle Park, North Carolina) accounting for sample weights and other design variables to generate nationally representative estimates.

## 3. Results

In 2009 and 2015, about 40% of adults were between the ages of 25 and 44 years old (42.0% in 2009 and 41.7% in 2015), and about half were male (49.9% in both 2009 and 2015). In addition, in 2009 and 2015, slightly over half resided in a rural area (50.2%, 53.4%), slightly less than one-fifth belonged to the lowest wealth index quintile (18.1%, 16.4%), and approximately 10% were unemployed or retired (9.5%, 9.8%). In 2009, nearly two-thirds (61.4%) of adults had completed secondary/high school or higher education; in 2015, nearly three-quarters (73.9%) of adults had achieved this level of education. In 2009, 28.2% of adults reported currently smoking tobacco; by 2015, the prevalence of current tobacco smoking decreased 5.6 percentage points to 22.7%. The percentage of adults who reported having a smoke-free policy at work increased by 11.0 percentage points (65.4% to 76.4%) from 2009 to 2015. Over 90% of adults believed SHS exposure causes serious illness in non-smokers in 2009 and 2015 (91.6% and 93.5%, respectively) (Table 1).

Overall, unadjusted exposure to SHS decreased from 2009 to 2015 across all five work and public places measured in the two survey waves: work (32.6% to 21.5%), government buildings (25.5% to 13.6%), healthcare facilities (7.6% to 4.2%), restaurants (33.6% to 21.9%), and public transportation (55.3% to 37.6%). The largest percentage point differences in SHS exposure between 2009 and 2015 were noted for public transportation; decreases ranged from 10.6 percentage points among adults who did not believe SHS exposure caused serious illness to 23.1 percentage points among adults who were unemployed or retired. In 2015, 63% of respondents who reported not having a smoke-free policy at their workplace were exposed to SHS at work, 44.2% on public transportation, almost 40% (37.8%) in restaurants, and nearly 30% (29.7%) in government buildings (Table 2).

In 2015, the unadjusted prevalence of SHS exposure in bars or nightclubs, universities, and schools or educational facilities varied across sociodemographic and other characteristics. In bars or nightclubs, SHS exposure ranged from 76.2% in adults who were unemployed or retired to 96.4% among those in the middle wealth index quintile. In universities, SHS exposure ranged from 5.1% in those with no formal or less than primary education to 20.5% in those whose workplaces had no smoke-free policy. In schools or educational facilities, SHS exposure ranged from 6.4% among those whose workplaces had a smoke-free policy to 19.4% among those who were unemployed or retired (Table 3).

After adjustment for all co-variates included in this study, the prevalence of SHS exposure decreased from 2009 to 2015 for all five work and public places measured in the two surveys. From 2009 to 2015, SHS exposure decreased 19% (or 5.7 percentage points) at work, 45% (or 11.2 percentage points) in government buildings, 48% (or 3.2 percentage points) in healthcare facilities, 29% (or 8.2 percentage points) in restaurants, and 33% (or 19.9 percentage points) on public transportation (Table 4).

## 4. Discussion

While SHS exposure varied by place, there was a significant decrease in the prevalence of SHS exposure at work and in the four other public places measured in both the 2009 and 2015 surveys (government buildings, healthcare facilities, restaurants, and public transportation). This was the case even after accounting for sociodemographic and other factors identified as being associated with SHS exposure in previous studies [23,24,25]. Similar to results reported by other studies, by 2015, less than 40% of adults were exposed to SHS on public transportation, approximately 20% in restaurants, less than 15% in government buildings, and less than 5% in healthcare facilities [19,26]. Also in 2015, nearly 90% of adults were exposed to SHS in bars or nightclubs, and approximately 10% reported SHS exposure in schools [19]. While tobacco control and prevention measures taken by the Philippine government may have helped reduce exposure to SHS between 2009 and 2015, this study highlights the importance of continued implementation, enforcement, monitoring, and evaluation of these measures.

Several notable tobacco control policies have been implemented in the Philippines which may have contributed to the decrease in SHS exposure observed in our study. Prior to the GATS conducted in the Philippines in 2009, the national Tobacco Regulation Act of 2003, in part, banned smoking in certain public places (e.g., schools and healthcare facilities) [11,12,14]. After the Philippines became a party to the WHO FCTC in 2005 [11,12], tobacco control efforts resulted in strengthened legislation and expanded regulations regarding smoking bans in public transportation and government buildings in 2009 [12]. Findings from the Baquilod et al., study, based on 2009 GATS data, showed that despite existing legislation at the time, there was a large proportion of adults exposed to SHS at work and in public [8]. This could, perhaps, have been due to non-compliance with and inadequate public awareness of existing policies, and thus there was an opportunity to improve the enforcement of smoke-free regulations [8].

Between 2009 and 2015 when the two waves of GATS were conducted, further tobacco control and prevention legislation was enacted in the form of additional tobacco tax increases [12,15]. Perhaps most significant was the adoption of the Sin Tax Reform Act in 2012 which increased excise taxes on tobacco products and shifted to a uniform tax rate for similar products [11,15]. The Sin Tax Reform Act resulted in major increases in the price of tobacco products; significantly increased tobacco tax revenues, part of which were earmarked for universal health coverage and supporting alternative livelihood programs for tobacco farmers and workers; and boosted the national health budget [15,27]. Smoking prevalence in the Philippines declined since the enforcement of the Sin Tax Reform Act in 2012 [15,26]. During this period, smoke-free policy enforcement by many local government units and various government agencies may also have been enhanced through competition for the Philippines Department of Health Red Orchid Awards launched in 2009 [16]. By reducing tobacco use, policies such as those which implement tax increases may also lead to decreases in SHS exposure, particularly among non-smokers [28].

We found a decrease in the adjusted prevalence of SHS exposure at work between 2009 and 2015; our 2009 estimates are similar to those reported elsewhere [8,25]. However, by 2015, almost one-quarter of adults who worked outside the home reported being exposed to SHS at work. Over the 2009–2015 period, the prevalence of adults who reported their workplace had a smoke-free policy increased by more than 10 percentage points (from 65.4% to 76.4%). Perhaps not surprisingly, in 2015, the highest proportion of adults exposed to SHS at work was among adults whose workplace did not have a comprehensive smoke-free policy. It was beyond the scope of our study to assess the characteristics of workplaces that did or did not have a smoke-free policy, or of their employees. However, previous research from Canada found that workers in certain occupations, such as trades, transport, and primary industry, were more often exposed to SHS at work, possibly due to challenges enforcing workplace smoke-free policies, unclear rules about where smoking is prohibited, or lack of existing legislation [29]. Additionally, a prior study using data from the Philippines found an inverse relationship between SHS exposure at work and socioeconomic status, particularly wealth and education [24]. The existence of socioeconomic inequalities in SHS exposure at work that we found may reflect differences in the implementation or enforcement of smoke-free policies across various types of employment. This highlights an opportunity to further decrease SHS exposure by encouraging the adoption, implementation, and enforcement of comprehensive smoke-free policies for all workplaces. A comprehensive smoke-free policy at work may encourage employees to adopt smoke-free rules at home [30].

We also found a substantial proportion of adults who used public transportation, visited restaurants, or visited bars or nightclubs reported exposure to SHS in those venues. The Philippine Land Transportation Franchising and Regulatory Board of the Department of Transportation and Communications Memorandum Circular No. 2009-036 banned smoking in all public utility vehicles (e.g., taxis, buses, etc.) and public land transportation terminals [12,31]. However, evidence from GATS Philippines 2015 showed that about 40% of adults reported SHS exposure on public transportation, indicating a need for increased enforcement to ensure compliance with the policy. Additionally, in 2015, about 20% of adults who visited restaurants and over 85% of adults who visited bars or nightclubs reported being exposed to SHS in such venues. Other than in food preparation areas, which by law must be smoke-free, restaurants, bars, and nightclubs in the Philippines may have designated smoking areas [12]. This may contribute to SHS exposure in these venues and suggests a need to consider enacting a comprehensive smoke-free policy for all public places in the Philippines.

Since 2015, the Philippines has continued to implement regulations that may further decrease SHS exposure, including the introduction of standards for designated smoking areas and imposing duties on people in charge of public places in 2017, further increases in excise taxes on cigarettes in 2019, and implementation and updating of regulations on packaging and labeling of tobacco products including graphic health warnings from 2016–2021 [12,15]. In 2020 and 2021, regulations and implementing rules were also introduced regarding the minimum sales age and graphic health warnings for e-cigarettes and heated tobacco products [12]. As of December 2021, smoking was prohibited in some indoor public places and workplaces (e.g., government facilities, healthcare facilities, and educational institutions) and most public transportation; however, designated smoking areas were still allowed in others (e.g., bars and nightclubs) [12]. Although beyond the scope of the current study, more recently SHS exposure may also have been affected by policy and behavioral changes associated with the COVID-19 pandemic as well as increases in the use of e-cigarettes and heated tobacco products; these are areas for future work. Thus, it is important to continue to monitor SHS exposure along with other tobacco-related indicators.

Our finding that, as of 2015, many adults in the Philippines are still exposed to SHS at work and in public places highlights the importance of the adoption of a comprehensive smoke-free policy for all work and public places. WHO policy recommendations to protect workers and the public from SHS exposure include enacting, implementing, and enforcing legislation that requires all indoor workplaces and public places to be 100% smoke-free [13]. Implementation of comprehensive smoke-free policies is effective for reducing SHS exposure, leads to reduced social inequalities in SHS exposure at work, and may also result in a reduction in smoking among those covered by the policies [2,32]. Evidence shows that smoke-free policies do not cause reduced business activity for bars and restaurants, and there is generally high public support for smoke-free public places and workplaces, which tends to increase following the implementation of smoke-free legislation [32]. The implementation of smoke-free policies may also lead to an interest in quitting and quit attempts; quit attempts may be supported through access to low-cost cessation medication [33]. Support for cessation through the Philippine Quit Line may also help those who have been encouraged to quit by providing free advice from counselors trained in supporting tobacco users to quit [34].

There are at least three limitations to our study. First, GATS data are self-reported and therefore subject to recall or social desirability biases of unknown direction. Second, GATS does not use biochemical markers to validate SHS exposure; however, previous research has found self-reported SHS exposure and biochemical markers to be correlated [35]. Third, we were not able to directly measure the impact of any one specific policy implemented by the Philippines using this data; this is an area of future work. Despite these limitations, to our knowledge, this is the only study conducted which assesses changes in SHS exposure over time in relation to the evolving tobacco control policy environment. Another strength of this study is the ability to generalize its findings as it was based on nationally representative GATS data from the Philippines.

## 5. Conclusions

We found a significant decrease in the prevalence of SHS exposure at work, in government buildings, healthcare facilities, restaurants, and public transportation in the Philippines from 2009 to 2015. However, a substantial proportion of adults is still exposed to SHS, particularly at work, in bars or nightclubs, on public transportation, and in restaurants, despite the many policy measures implemented over time. This study highlights the importance of continued implementation, enforcement, monitoring, and evaluation of measures taken to reduce exposure to SHS, including comprehensive smoke-free policies. Using data, governmental and non-governmental organizations can work together to utilize policies to effectively reduce tobacco use and SHS exposure and to create a healthier environment for everyone.

## Figures and Tables

**Table 1:** Sociodemographic characteristics, tobacco use, and SHS policies among adults aged 15 years or older in the Philippines, 2009 and 2015.

Characteristics		2009		2015		Percentage Point Difference ^1^
		n	% (95% CI)	n	% (95% CI)	
Age group (years)	15–24 years	1988	29.6 (28.4–30.8)	2338	29.4 (28.3–30.6)	−0.2
	25–44 years	4581	42.0 (40.7–43.3)	4967	41.7 (40.6–42.8)	−0.3
	45–64 years	2360	22.0 (21.0–23.0)	3210	22.4 (21.6–23.3)	0.4
	65 years or older	772	6.5 (5.9–7.1)	1129	6.5 (6.0–7.0)	0.1
Sex	Male	4740	49.9 (49.0–50.8)	5781	49.9 (48.8–51.0)	0.0
	Female	4961	50.1 (49.2–51.0)	5863	50.1 (49.0–51.2)	0.0
Residence	Urban	4332	49.8 (47.6–51.9)	4610	46.6 (42.5–50.7)	−3.2
	Rural	5369	50.2 (48.1–52.4)	7034	53.4 (49.3–57.5)	3.2
Wealth index (quintiles)	Lowest	1966	18.1 (16.4–20.0)	2600	16.4 (15.0–18.1)	−1.7
	Low	1897	18.1 (17.0–19.3)	2235	17.5 (16.5–18.5)	−0.6
	Middle	1960	20.7 (19.5–21.9)	2167	18.8 (17.9–19.8)	−1.9 ^2^
	High	1939	20.4 (19.3–21.5)	2314	21.8 (20.7–23.0)	1.4
	Highest	1939	22.7 (21.0–24.5)	2328	25.5 (23.9–27.1)	2.8 ^2^
Education	No formal education/less than primary	1900	21.5 (19.9–23.0)	2132	14.4 (13.3–15.6)	−7.1 ^2^
	Completed primary/less than secondary	1371	17.2 (16.0–18.4)	1571	11.7 (10.9–12.6)	−5.5 ^2^
	Completed secondary/completed high school	3987	39.1 (37.7–40.6)	4984	45.0 (43.7–46.4)	5.9 ^2^
	Completed college/university or above	2442	22.3 (20.8–23.8)	2953	28.9 (27.4–30.4)	6.6 ^2^
Occupation	Employed	3224	35.2 (33.7–36.8)	3563	34.4 (32.9–35.9)	−0.8
	Self-employed	3066	27.5 (26.1–29.0)	3773	26.5 (25.2–27.8)	−1.1
	Student	713	10.7 (9.8–11.7)	972	12.2 (11.4–13.1)	1.5 ^2^
	Homemaker	1857	17.0 (16.0–18.0)	2192	17.1 (16.3–18.1)	0.1
	Unemployed/retired	839	9.5 (8.6–10.4)	1104	9.8 (8.9–10.8)	0.3
Current tobacco smoker	Yes	2769	28.2 (27.0–29.5)	2793	22.7 (21.6–23.7)	−5.6 ^2^
	No	6932	71.8 (70.5–73.0)	8851	77.3 (76.3–78.4)	5.6 ^2^
Smoke-free policy at work	Smoke-free policy	1676	65.4 (62.6–68.1)	2234	76.4 (73.8–78.8)	11.0 ^2^
	No smoke-free policy	983	34.6 (31.9–37.4)	810	23.6 (21.2–26.2)	−11.0 ^2^
Believes SHS exposure causes serious illness in non-smokers	Yes	8916	91.6 (90.7–92.5)	10,811	93.5 (92.4–94.5)	1.9 ^2^
	No	783	8.4 (7.5–9.3)	829	6.5 (5.5–7.6)	−1.9 ^2^

Abbreviations: SHS, secondhand smoke; CI, confidence interval; ^1^ Percentage point difference is calculated as the 2015 percentage minus the 2009 percentage, and may not match exactly due to rounding; ^2^ T-test comparing the difference between 2015 and 2009 was significant at *p*-value < 0.05.

**Table 2:** Prevalence of SHS exposure by sociodemographic characteristics, tobacco use, and smoking policies among adults aged 15 years or older in the Philippines, 2009 and 2015.

		SHS Exposure at Work ^1^			SHS Exposure in Government Buildings ^2^			SHS Exposure in Healthcare Facilities ^2^			SHS Exposure in Restaurants ^2^			SHS Exposure on Public Transportation ^2^		
Variable		2009 (*n* = 785)	2015 (*n* = 604)	Percentage Point Difference (PPD) ^3^	2009 (*n* = 1070)	2015 (*n* = 665)	PPD ^3^	2009 (*n* = 278)	2015 (*n* = 217)	PPD ^3^	2009 (*n* = 1686)	2015 (*n* = 1312)	PPD ^3^	2009 (*n* = 4240)	2015 (*n* = 3152)	PPD ^3^
		% (95% CI)	% (95% CI)		% (95% CI)	% (95% CI)		% (95% CI)	% (95% CI)		% (95% CI)	% (95% CI)		% (95% CI)	% (95% CI)	
Overall		32.6 (29.9–35.5)	21.5 (19.1–24.0)	−11.2 ^4^	25.5 (23.3–27.8)	13.6 (11.9–15.5)	−11.9 ^4^	7.6 (6.5–8.9)	4.2 (3.6–5.0)	−3.4 ^4^	33.6 (31.2–36.1)	21.9 (20.1–23.8)	−11.7 ^4^	55.3 (53.3–57.3)	37.6 (35.6–39.6)	−17.7 ^4^
Age group (years)	15–24 years	30.5 (25.2–36.5)	17.9 (13.9–22.8)	−12.6 ^4^	25.6 (21.7–29.9)	12.3 (9.8–15.3)	−13.3 ^4^	9.2 (6.8–12.4)	3.8 (2.6–5.6)	−5.4 ^4^	33.8 (30.1–37.7)	20.0 (17.2–23.0)	−13.9 ^4^	57.4 (54.4–60.4)	38.1 (35.1–41.2)	−19.3 ^4^
	25–44 years	30.4 (27.3–33.7)	22.0 (19.2–25.2)	−8.4 ^4^	25.2 (22.8–27.9)	13.5 (11.2–16.2)	−11.7 ^4^	6.9 (5.4–8.7)	4.2 (3.3–5.2)	−2.7 ^4^	35.1 (32.3–38.0)	23.2 (20.8–25.8)	−11.9 ^4^	56.6 (53.9–59.2)	39.3 (36.8–41.9)	−17.3 ^4^
	45–64 years	37.9 (32.7–43.4)	23.2 (19.6–27.1)	−14.8 ^4^	25.4 (22.2–28.9)	14.5 (12.3–17.1)	−10.8 ^4^	7.0 (5.3–9.2)	4.5 (3.4–5.9)	−2.6 ^4^	30.1 (26.4–34.1)	22.9 (20.2–25.8)	−7.3 ^4^	52.1 (49.1–55.1)	35.4 (32.7–38.2)	−16.7 ^4^
	65 years or older	56.9 (41.4–71.1)	30.6 (20.5–43.0)	−26.3 ^4^	27.8 (20.9–35.9)	15.9 (11.2–22.1)	−11.9 ^4^	7.7 (4.2–13.5)	5.3 (3.1–8.9)	−2.3	32.4 (24.7–41.1)	18.4 (13.7–24.4)	−13.9 ^4^	45.0 (38.9–51.2)	30.8 (26.8–35.1)	−14.2 ^4^
Sex	Male	38.8 (35.1–42.7)	26.4 (23.3–29.8)	−12.4 ^4^	27.9 (25.3–30.7)	15.6 (13.4–18.2)	−12.3 ^4^	8.0 (6.4–10.0)	4.9 (3.9–6.3)	−3.1 ^4^	38.4 (35.3–41.5)	26.8 (24.3–29.4)	−11.6 ^4^	61.1 (58.6–63.5)	39.9 (37.5–42.5)	−21.1 ^4^
	Female	26.2 (22.9–29.8)	16.4 (13.5–19.8)	−9.8 ^4^	23.1 (20.6–25.9)	11.7 (9.9–13.7)	−11.5 ^4^	7.3 (6.0–8.9)	3.8 (3.0–4.7)	−3.6 ^4^	28.6 (26.0–31.4)	17.0 (15.1–19.0)	−11.7 ^4^	49.7 (47.3–52.1)	35.5 (33.3–37.7)	−14.2 ^4^
Residence	Urban	25.3 (22.2–28.6)	18.2 (15.3–21.7)	−7.0 ^4^	23.7 (21.0–26.7)	13.7 (10.8–17.3)	−10.0 ^4^	7.9 (6.3–9.8)	3.7 (2.8–4.8)	−4.2 ^4^	27.0 (24.2–29.9)	19.2 (16.8–21.9)	−7.8 ^4^	59.4 (56.5–62.2)	40.6 (37.8–43.5)	−18.8 ^4^
	Rural	46.2 (41.8–50.7)	26.8 (23.4–30.6)	−19.4 ^4^	27.1 (23.9–30.6)	13.5 (11.6–15.6)	−13.7 ^4^	7.3 (5.8–9.1)	4.7 (3.8–5.8)	−2.6 ^4^	41.9 (38.2–45.7)	25.1 (22.4–27.9)	−16.9 ^4^	50.7 (48.0–53.4)	34.5 (31.9–37.3)	−16.2 ^4^
Wealth index (quintiles)	Lowest	53.3 (43.8–62.5)	30.7 (23.3–39.2)	−22.6 ^4^	24.1 (19.2–29.7)	14.4 (11.4–18.0)	−9.7 ^4^	7.2 (5.0–10.3)	4.3 (3.0–6.2)	−2.8	46.2 (39.3–53.3)	21.9 (17.9–26.5)	−24.4 ^4^	50.4 (45.9–55.0)	31.4 (27.6–35.6)	−19.0 ^4^
	Low	39.0 (32.0–46.5)	28.8 (22.6–35.8)	−10.3 ^4^	25.2 (21.3–29.5)	13.2 (10.5–16.5)	−11.9 ^4^	7.6 (5.4–10.7)	6.0 (4.2–8.5)	−1.6	39.9 (35.1–45.0)	26.9 (22.9–31.4)	−13.0 ^4^	52.8 (49.3–56.3)	34.8 (31.6–38.1)	−18.0 ^4^
	Middle	33.6 (28.2–39.4)	22.7 (18.3–27.9)	−10.9 ^4^	23.2 (19.5–27.3)	14.1 (10.3–19.0)	−9.1 ^4^	7.8 (5.8–10.4)	3.6 (2.4–5.3)	−4.2 ^4^	35.2 (31.0–39.6)	23.1 (19.9–26.8)	−12.0 ^4^	54.0 (50.4–57.6)	37.4 (34.2–40.7)	−16.7 ^4^
	High	30.3 (25.4–35.6)	20.2 (16.5–24.3)	−10.1 ^4^	27.7 (24.0–31.9)	14.3 (11.6–17.4)	−13.5 ^4^	5.3 (3.7–7.5)	4.2 (3.0–5.9)	−1.0	33.2 (29.4–37.3)	21.6 (18.7–24.7)	−11.7 ^4^	56.4 (53.1–59.6)	38.2 (35.2–41.3)	−18.2 ^4^
	Highest	26.2 (22.3–30.4)	17.3 (14.0–21.3)	−8.8 ^4^	26.4 (22.9–30.3)	12.5 (10.2–15.3)	−13.9 ^4^	9.7 (7.3–12.9)	3.5 (2.5–4.9)	−6.2 ^4^	25.5 (22.3–28.9)	19.7 (17.2–22.5)	−5.8^4^	60.5 (57.1–63.7)	42.4 (39.4–45.6)	−18.0 ^4^
Education	No formal education/less than primary	59.2 (50.6–67.3)	39.9 (32.3–48.0)	−19.3 ^4^	24.3 (19.7–29.5)	16.1 (12.4–20.7)	−8.2 ^4^	6.5 (4.5–9.3)	5.4 (3.6–8.0)	−1.1	45.7 (39.6–51.9)	26.1 (21.5–31.3)	−19.6 ^4^	49.3 (45.4–53.2)	31.1 (27.6–34.9)	−18.2 ^4^
	Completed primary/less than secondary	45.1 (36.1–54.4)	35.7 (27.6–44.7)	−9.4	23.4 (19.0–28.3)	14.8 (11.5–18.8)	−8.6 ^4^	7.0 (4.5–10.7)	4.0 (2.5–6.4)	−3.0	32.8 (27.5–38.6)	25.6 (21.2–30.5)	−7.3	49.7 (45.6–53.7)	36.5 (32.7–40.5)	−13.2 ^4^
	Completed secondary/completed high school	30.8 (26.8–35.0)	22.8 (19.5–26.4)	−8.0 ^4^	24.7 (21.9–27.9)	14.0 (11.5–17.1)	−10.7 ^4^	7.4 (5.8–9.5)	4.3 (3.3–5.5)	−3.1 ^4^	33.1 (30.0–36.4)	22.4 (20.2–24.9)	−10.7 ^4^	56.6 (53.9–59.3)	38.0 (35.6–40.5)	−18.5 ^4^
	Completed college/university or above	23.7 (20.4–27.3)	16.2 (13.5–19.4)	−7.5 ^4^	28.2 (24.8–32.0)	11.8 (9.9–14.1)	−16.4 ^4^	8.8 (6.9–11.2)	3.7 (2.7–5.0)	−5.1 ^4^	29.1 (26.2–32.3)	19.8 (17.5–22.2)	−9.4 ^4^	61.6 (58.4–64.7)	40.0 (37.1–43.0)	−21.6 ^4^
Occupation	Employed	25.9 (23.1–28.9)	18.2 (15.9–20.7)	−7.7 ^4^	24.1 (21.3–27.2)	13.7 (11.4–16.5)	−10.4 ^4^	7.2 (5.3–9.6)	3.2 (2.4–4.4)	−3.9 ^4^	32.0 (28.7–35.4)	21.4 (19.0–24.1)	−10.5 ^4^	56.8 (53.9–59.6)	39.0 (36.3–41.7)	−17.8 ^4^
	Self-employed	55.7 (49.9–61.3)	33.8 (28.9–39.0)	−21.9 ^4^	27.7 (24.5–31.1)	13.3 (11.0–16.0)	−14.4 ^4^	6.9 (5.3–9.0)	4.9 (3.7–6.5)	−2.0	39.1 (35.4–43.0)	26.3 (23.5–29.2)	−12.9 ^4^	53.0 (50.0–56.0)	36.9 (34.0–39.8)	−16.1 ^4^
	Student	52.6 (28.1–76.0)	25.1 (11.0–47.7)	−27.5	29.1 (23.4–35.6)	9.5 (6.7–13.4)	−19.6 ^4^	8.6 (4.9–14.6)	4.2 (2.4–7.4)	−4.4	30.0 (25.1–35.3)	22.1 (18.3–26.5)	−7.8 ^4^	60.3 (55.7–64.7)	39.4 (35.4–43.5)	−20.9 ^4^
	Homemaker	44.6 (19.3–73.1)	27.9 (17.8–40.8)	−16.8	24.4 (20.5–28.7)	15.0 (11.9–18.8)	−9.4 ^4^	6.7 (4.9–9.3)	4.5 (3.3–6.1)	−2.3	31.2 (26.6–36.2)	17.5 (14.4–21.1)	−13.7 ^4^	49.0 (45.0–52.9)	34.0 (30.7–37.4)	−15.0 ^4^
	Unemployed/retired	49.6 (19.0–80.4)	42.2 (17.7–71.3)	−7.3	21.1 (15.5–28.2)	16.2 (11.9–21.6)	−4.9	12.2 (8.0–18.0)	5.3 (3.2–8.7)	−6.8 ^4^	33.4 (26.8–40.6)	17.9 (13.8–22.8)	−15.5 ^4^	61.1 (56.2–65.7)	38.0 (33.2–42.9)	−23.1 ^4^
Current tobacco smoker	Yes	46.2 (40.9–51.7)	34.2 (29.2–39.5)	−12.0 ^4^	26.6 (23.3–30.2)	18.4 (15.5–21.8)	−8.2 ^4^	7.3 (5.3–9.9)	6.1 (4.3–8.5)	−1.2	40.7 (36.9–44.6)	27.7 (24.1–31.5)	−13.0 ^4^	59.6 (56.5–62.6)	39.1 (35.9–42.3)	−20.5 ^4^
	No	28.0 (25.2–31.1)	17.8 (15.4–20.5)	−10.2 ^4^	25.1 (22.8–27.5)	12.2 (10.5–14.1)	−12.9 ^4^	7.7 (6.4–9.2)	3.8 (3.2–4.7)	−3.8 ^4^	31.1 (28.6–33.7)	20.3 (18.5–22.3)	−10.8 ^4^	53.6 (51.4–55.8)	37.2 (35.1–39.3)	−16.4 ^4^
Smoke-free policy at work	Smoke-free policy	12.7 (10.7–15.1)	9.8 (8.3–11.6)	−2.9 ^4^	22.9 (19.2–27.0)	9.3 (7.4–11.7)	−13.5 ^4^	6.0 (3.8–9.3)	2.5 (1.6–3.8)	−3.5 ^4^	24.8 (21.4–28.6)	14.8 (12.7–17.3)	−10.0 ^4^	57.9 (54.4–61.4)	38.4 (35.3–41.5)	−19.6 ^4^
	No smoke-free policy	77.4 (73.2–81.2)	63.0 (58.1–67.7)	−14.4 ^4^	32.8 (27.3–38.8)	29.7 (23.6–36.5)	−3.1	8.1 (5.6–11.7)	6.0 (3.7–9.5)	−2.2	39.5 (34.3–45.0)	37.8 (31.9–44.0)	−1.8	63.5 (59.1–67.7)	44.2 (39.0–49.5)	−19.4 ^4^
Believes SHS exposure causes serious illness in non-smokers	Yes	31.7 (29.0–34.6)	21.1 (18.7–23.6)	−10.7 ^4^	25.6 (23.4–27.9)	13.0 (11.5–14.6)	−12.6 ^4^	7.5 (6.4–8.8)	4.1 (3.5–4.9)	−3.4 ^4^	33.0 (30.6–35.4)	21.9 (20.1–23.9)	−11.0 ^4^	55.7 (53.6–57.8)	37.5 (35.5–39.5)	−18.2 ^4^
	No	50.9 (38.5–63.2)	30.6 (20.3–43.3)	−20.3 ^4^	24.0 (18.0–31.2)	25.4 (14.2–41.2)	1.4	9.8 (5.5–17.0)	6.6 (3.9–11.1)	−3.2	43.6 (35.1–52.4)	21.6 (15.4–29.3)	−22.0 ^4^	50.0 (44.6–55.4)	39.4 (31.7–47.7)	−10.6 ^4^

Abbreviations: SHS, secondhand smoke; CI, confidence interval; PPD, percentage point difference; ^1^ Adults who worked outside the home, either indoors or outdoors, and were exposed to tobacco smoke in indoor areas at work during the past 30 days; ^2^ Adults who visited in the past 30 days and were exposed to tobacco smoke inside; ^3^ Percentage point difference is calculated as the 2015 percentage minus the 2009 percentage and may not match exactly due to rounding; ^4^ T-test comparing the difference between 2015 and 2009 was significant at *p*-value < 0.05.

**Table 3:** Prevalence of SHS exposure by sociodemographic characteristics, tobacco use, and smoking policies among adults aged 15 years or older in the Philippines, 2015.

		SHS Exposure in Bars or Night Clubs ^1^	SHS Exposure in Universities ^1^	SHS Exposure in Schools or Educational Facilities ^1^
Variable		2015 (*n* = 487)	2015 (*n* = 197)	2015 (*n* = 455)
		% (95% CI)	% (95% CI)	% (95% CI)
Overall		86.3 (82.0–89.7)	15.1 (12.6–18.1)	10.9 (9.6–12.4)
Age group (years)	15–24 years	84.3 (74.5–90.8)	17.2 (13.5–21.6)	16.3 (13.7–19.3)
	25–44 years	87.4 (81.9–91.4)	14.3 (10.9–18.7)	8.2 (6.8–9.8)
	45–64 years	87.2 (74.6–94.0)	9.9 (5.8–16.5)	8.5 (6.5–11.1)
	65 years or older	—^2^	6.5 (2.3–17.0)	8.1 (4.9–13.1)
Sex	Male	88.9 (84.2–92.3)	15.8 (12.4–19.9)	12.7 (10.7–15.0)
	Female	78.9 (69.1–86.2)	14.5 (11.4–18.4)	9.6 (8.1 –11.4)
Residence	Urban	84.1 (78.0–88.7)	13.0 (9.9–17.0)	10.1 (8.2–12.6)
	Rural	90.5 (85.6–93.9)	17.8 (13.8–22.7)	11.5 (9.8–13.5)
Wealth index (quintiles)	Lowest	87.5 (74.5–94.4)	19.0 (10.7–31.3)	9.6 (7.4–12.3)
	Low	91.5 (81.2–96.4)	12.2 (7.7–18.7)	11.7 (9.0–15.1)
	Middle	96.4 (91.1–98.6)	14.2 (9.3–21.1)	11.8 (9.0–15.2)
	High	83.8 (74.5–90.2)	12.7 (9.0–17.7)	10.2 (8.0–12.9)
	Highest	83.0 (76.2–88.2)	17.0 (13.3–21.6)	11.3 (9.0–14.1)
Education	No formal education/less than primary	88.7 (75.7–95.2)	5.1 (1.3–17.4)	8.2 (5.9–11.1)
	Completed primary/less than secondary	91.5 (79.5–96.8)	14.6 (7.0–27.9)	9.7 (6.7–13.9)
	Completed secondary/completed high school	90.4 (82.1–95.1)	13.5 (10.4–17.5)	12.1 (10.3–14.1)
	Completed college/university or above	82.4 (76.2–87.2)	16.7 (13.3–20.8)	10.6 (8.3–13.4)
Occupation	Employed	87.7 (82.6–91.5)	14.2 (10.4–19.0)	8.7 (6.9–11.0)
	Self-employed	86.2 (78.4–91.5)	9.6 (5.9–15.3)	7.8 (6.2–9.7)
	Student	86.7 (71.9–94.3)	17.5 (13.5–22.5)	17.0 (13.7–20.9)
	Homemaker	85.9 (69.7–94.1)	16.4 (10.1–25.6)	8.9 (6.8–11.7)
	Unemployed/retired	76.2 (51.4–90.6)	15.7 (8.7–26.7)	19.4 (13.8–26.6)
Current tobacco smoker	Yes	93.1 (89.1–95.6)	16.4 (11.3–23.2)	10.9 (8.3–14.1)
	No	80.7 (74.0–86.1)	14.9 (12.2–18.1)	10.9 (9.4–12.6)
Smoke-free policy at work	Smoke-free policy	84.5 (77.5–89.6)	11.0 (7.2–16.4)	6.4 (4.6–9.0)
	No smoke-free policy	90.0 (80.6–95.2)	20.5 (11.5–33.8)	12.6 (8.4–18.5)
Believes SHS exposure causes serious illness in non-smokers	Yes	85.9 (81.5–89.4)	15.1 (12.5–18.2)	10.8 (9.5–12.3)
	No	— ^2^	15.8 (7.1–31.5)	14.2 (8.9–21.9)

Abbreviations: SHS, secondhand smoke; CI, confidence interval; PPD, percentage point difference; ^1^ Adults who visited in the past 30 days and were exposed to tobacco smoke inside; ^2^ Estimate suppressed due to unweighted denominator less than 25.

**Table 4:** Adjusted prevalence ^1^ of SHS exposure by sociodemographic characteristics, tobacco use, and smoking policies among adults aged 15 years or older in the Philippines, 2009 and 2015.

	2009	2015	2015 vs. 2009	
	Adjusted % (95% CI)	Adjusted % (95% CI)	APR (95% CI)	Adjusted Prevalence Difference (95% CI)
SHS exposure at work ^2^	29.5 (27.2–31.9)	23.7 (21.6–26.0)	0.81 (0.73–0.89)	−5.7 (−8.3 to −3.2) ^4^
SHS exposure in government buildings ^3^	25.3 (22.2–28.6)	14.0 (11.7–16.7)	0.55 (0.45–0.69)	−11.2 (−15.3 to −7.2) ^4^
SHS exposure in healthcare facilities ^3^	6.6 (4.8–9.0)	3.4 (2.5–4.7)	0.52 (0.33–0.81)	−3.2 (−5.5 to −0.9) ^4^
SHS exposure in restaurants ^3^	28.4 (25.3–31.6)	20.2 (17.9–22.8)	0.71 (0.61–0.84)	−8.2 (−12.1 to −4.3) ^4^
SHS exposure on public transportation ^3^	59.7 (56.8–62.5)	39.8 (37.2–42.5)	0.67 (0.62–0.72)	−19.9 (−23.7 to −16.1) ^4^

Abbreviations: SHS, secondhand smoke; CI, confidence interval; APR, adjusted prevalence ratio; ^1^ Adjusted for: age, sex, residence, wealth index, education, occupation, tobacco use, smoking policy at work, and belief that SHS causes series illness in non-smokers; ^2^ Adults who worked outside the home, either indoors or outdoors, and were exposed to tobacco smoke in indoor areas at work during the past 30 days; ^3^ Adults who visited in the past 30 days and were exposed to tobacco smoke inside; ^4^ Adjusted prevalence difference between 2015 and 2009 was statistically significant at *p*-value < 0.05.

## Data Availability

Publicly available datasets were analyzed in this study. This data can be found here: https://www.cdc.gov/tobacco/global/gtss/gtssdata/index.html (accessed on 29 June 2022).
